# Noise Pollution Filters Bird Communities Based on Vocal Frequency

**DOI:** 10.1371/journal.pone.0027052

**Published:** 2011-11-09

**Authors:** Clinton D. Francis, Catherine P. Ortega, Alexander Cruz

**Affiliations:** 1 Ecology and Evolutionary Biology, University of Colorado, Boulder, Colorado, United States of America; 2 San Juan Institute of Natural and Cultural Resources, Fort Lewis College, Durango, Colorado, United States of America; University of Bristol, United Kingdom

## Abstract

**Background:**

Human-generated noise pollution now permeates natural habitats worldwide, presenting evolutionarily novel acoustic conditions unprecedented to most landscapes. These acoustics not only harm humans, but threaten wildlife, and especially birds, via changes to species densities, foraging behavior, reproductive success, and predator-prey interactions. Explanations for negative effects of noise on birds include disruption of acoustic communication through energetic masking, potentially forcing species that rely upon acoustic communication to abandon otherwise suitable areas. However, this hypothesis has not been adequately tested because confounding stimuli often co-vary with noise and are difficult to separate from noise exposure.

**Methodology/Principal Findings:**

Using a natural experiment that controls for confounding stimuli, we evaluate whether species vocal features or urban-tolerance classifications explain their responses to noise measured through habitat use. Two data sets representing nesting and abundance responses reveal that noise filters bird communities nonrandomly. Signal duration and urban tolerance failed to explain species-specific responses, but birds with low-frequency signals that are more susceptible to masking from noise avoided noisy areas and birds with higher frequency vocalizations remained. Signal frequency was also negatively correlated with body mass, suggesting that larger birds may be more sensitive to noise due to the link between body size and vocal frequency.

**Conclusions/Significance:**

Our findings suggest that acoustic masking by noise may be a strong selective force shaping the ecology of birds worldwide. Larger birds with lower frequency signals may be excluded from noisy areas, whereas smaller species persist via transmission of higher frequency signals. We discuss our findings as they relate to interspecific relationships among body size, vocal amplitude and frequency and suggest that they are immediately relevant to the global problem of increases in noise by providing critical insight as to which species traits influence tolerance of these novel acoustics.

## Introduction

Anthropogenic noise pollution (hereafter “noise”) now permeates natural areas worldwide, and these evolutionarily novel acoustics are not only problematic for human wellbeing [1], [2], but negatively affect bird distributions, community diversity and predator-prey interactions [3]–[6]. A likely cause for declines in bird distributions in noisy areas is because noise interferes with vocal communication, whereby birds with low-frequency vocalizations may be unable to communicate in the presence of low-frequency industrial noise and must abandon otherwise suitable areas [7]–[9]. This explanation is supported by the observation that urban-tolerant species may be predisposed to occupy noisy urban areas because they have higher frequency signals that may suffer less acoustic interference from urban noise than birds that vocalize at lower frequencies [10]. Yet because urban-tolerant birds have broader environmental tolerances than non-urban birds [11] and because their occupancy of urban environments also depends on key foraging and nesting opportunities [12], it is not yet clear whether urban-tolerant species persist in urban areas due to their signaling characteristics or because of other factors.

Outside of urban areas, several studies have suggested that a likely cause for declines in bird abundances in response to traffic noise is because noise masks vocal communication (e.g. refs. [7], [13], [14]), yet these studies have not adequately linked declines in bird abundances to interference with acoustic communication for several reasons (reviewed in refs. [5], [9]). First, several stimuli that often co-vary with noise could also explain the declines, such as edge habitat, vehicular motion and lights, or direct mortality of birds due to collisions with vehicles. Second, the presence of noise can also severely impair an observer's ability to detect birds [15], [16], biasing surveys used to determine whether species distributions are noise-dependent. Finally, although frequency may be one feature that influences signal transmission in noisy conditions, other signal features, such as greater signal duration, may also improve signal detection in noisy areas [17]–[19] and should be considered when evaluating species-specific responses to noise.

**Figure 1:**
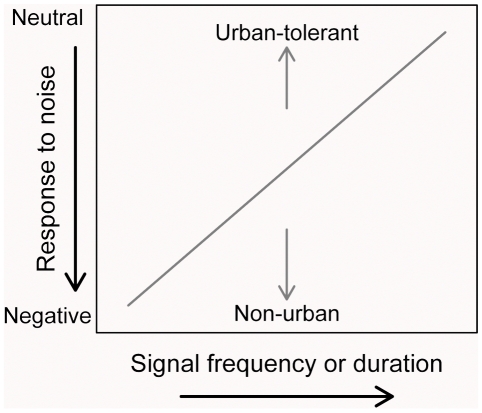
Predicted influences of vocal features and urban-tolerance classifications on species-specific responses to noise. If the degree to which species can successfully dispatch and receive acoustic signals influences their distributions in environments characterized by anthropogenic noise, species that have high-frequency vocalizations or long signal durations may have a neutral response to noise, but species that vocalize at low frequencies or with short signals may avoid noisy areas. Similarly, if urban birds have signals predisposed to noisy urban areas, urban-tolerant species should have neutral to marginally negative responses to noise compared to strong negative responses by non-urban species, even in noisy non-urban areas.

Here, we used a unique study system that separates noise from confounding stimuli to evaluate which species' traits predict species-specific sensitivities to noise as measured by habitat use. If species distributions in noisy environments are determined by their ability to communicate, then species with signals that are higher in frequency and/or longer in duration should be less sensitive, and their distributions should change little between noisy and quiet environments. In contrast, species with signals that are lower frequency signals and/or shorter in duration should be more sensitive and should be more common in quiet relative to noisy areas (Fig. 1). These signaling features may also influence urban tolerance and explain why urban-tolerant birds persist in noisy cities [10]; however, tolerance to a broad range of environmental conditions and exploitation of key foraging and nesting opportunities could also explain the persistence of urban-tolerant species in cities [11], [12]. If persistence in noisy cities is linked to signaling features, then urban-tolerant species should be less sensitive to noise than non-urban species in their habitat use in noisy non-urban habitats (Fig. 1). In contrast, if persistence in noisy cities is linked to exploitation of other key features within urban areas, urban-tolerant and non-urban birds should not differ in their habitat use in noisy non-urban habitats. In attempt to tease apart these influences, we first determine whether urban tolerance or signaling features explain habitat use in response to noise. We then test whether urban-tolerant birds differ in vocal features from non-urban birds, and we explore how vocal features relate to body size.

## Methods

This study was completed in compliance with the University of Colorado Animal Care and Use Guidelines. The University of Colorado's Animal Care and Use Committee reviewed the study's methods and determined it did not need IACUC approval because the methods were observational only.

### Study area

**Figure 2:**
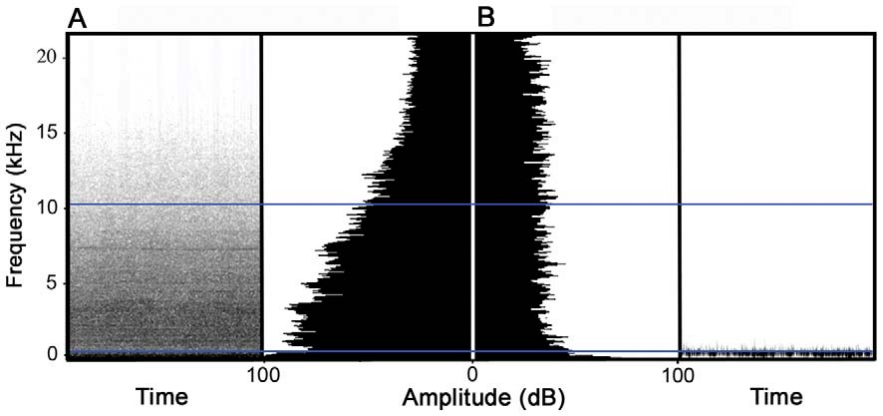
Examples of background noise on a noisy treatment (A) and quiet control site (B). Spectrograms are on the outside panels and power spectra are located on the center panels. Darker shades in spectrograms indicate more acoustic energy located at those frequencies, which is reflected by higher amplitude values in the power spectra. On noisy treatment sites, acoustic energy from compressors increases at lower frequencies and represents a greater masking potential for species with low-frequency vocalizations. This masking potential is absent on quiet control sites. Horizontal lines denote approximate minimum and maximum vocal frequencies of birds considered in this study (see also Audio S1 and S2 for sample recordings of background noise on treatment and control sites).

We conducted our study within Rattlesnake Canyon Habitat Management Area (RCHMA), which is located in the San Juan Basin in northwestern New Mexico and managed by the Bureau of Land Management (BLM). RCHMA is dominated by piñon (*Pinus edulis*)-juniper (*Juniperus osteosperma*) woodlands and is within one of the United States' most developed energy-producing regions (over 20,000 active oil and gas wells within the San Juan Basin). Gas wells are often coupled with compressors, which aid in the extraction and transportation of gas through pipelines and run 24 hours a day, 365 days a year aside from periodic maintenance and our bird surveys and nest searches [6], [19], . Similar to most anthropogenic noise, compressor noise has most energy at low frequencies and gradually diminishes towards higher frequencies; thus, the energetic masking potential by compressor noise progressively increases for lower frequency signals (Fig. 2; for details on compressor noise see refs. [6], [20]). Noisy compressors are present on some well pads (treatment sites) and absent on others (control sites), which provides a unique opportunity to determine the influence of noise on natural populations and communities. Critical to this design, with the exception of background noise amplitude, which is significantly higher on treatment sites than control sites through a distance of 400 m from the compressor or wellhead, human activity and vegetation did not to differ on and around the well pads with and without noisy compressors that were used in this study [6]; thus, effects of noise are separated from other confounding variables that complicated previous attempts to characterize the influence of noise on bird distributions [7], [13], [14]. Finally, and perhaps most critically, compressors were turned off during our visits to noisy treatment sites to quantify responses to noise (see below) to control for the negative influence of noise on observers' abilities to detect birds [15].

### Responses to noise

We searched for and monitored nests at nine treatment and nine control sites during the breeding seasons of 2005 and 2006 and ten treatment and eight control sites in 2007. In 2007, we also conducted point count surveys at eight control sites and five treatment sites with compressors turned off during our surveys on treatment sites. Methodological details for nest searching and monitoring, plus the bird surveys can be found elsewhere [6], [20]. Hereafter we refer to “nesting response” when referring to the nesting data and “abundance response” for the survey data. From the surveys, at each location we estimated a species abundance as the maximum number of individuals detected during one of two visits rather than summing the total from both visits, which would have double-counted individuals that were detected on both the first and second visit. Additionally, because of increases in identification error with distance, we restricted our abundance estimates to only those individuals observed within 60 m from the point count location.

We estimated the nesting response to noise as the ratio of the mean number of nests per treatment and control site:
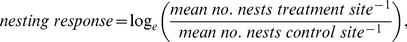
(1)Prior to calculating the mean number of nests per site, we performed a quantitative adjustment to the data by adding one to the total number of nests detected on treatment sites and to the total number of nests detected on control sites. This was necessary because some species did not nest on one of the two site types, precluding our ability to gauge response to noise as a ratio.

Abundance response to noise was estimated as the ratio of the mean number of individuals per survey location on treatment and control sites:
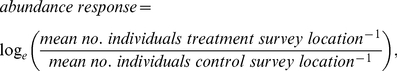
(2)Subsequently, these ratios represent the relative strength of the response of each species to noise in terms of habitat use.

### Species vocalization features and urban tolerance

Vocalizations of all species were recorded at sites in our study area between 11 May and 2 July 2009. To ensure for independence of samples, we only sampled one individual per species at each site, or for the minority of occasions when we did sample more than one individual per species on a site, we only sampled individuals that maintained non-adjacent territories. We also recorded vocalizations from individuals located in noisy and quiet areas to capture potential vocal variation among individuals in quiet and noisy areas (e.g., refs. [8], [19], [20]).

We recorded vocalizations with a Marantz PMD 660 Digital recorder using a directional shotgun microphone (Audio-technica AT-815) pointed directly at the vocalizing individual (WAV format, sampling rate = 48 kHz, bitrate = 1536 kbps). We recorded vocalizations for entire song or call bouts (i.e. duration that an individual vocalizes from a single perch) when wind speed was less than category three (≈13–18 kmh^−1^) on the Beaufort Wind Scale.

For each individual recorded, we randomly selected five strophes or calls from each recording and measured the following variables: vocalization length, number of notes, minimum and maximum frequency, peak frequency (the frequency vocalized at the highest amplitude), and peak frequency of the lowest note (highest amplitude of the call or song's lowest note). Peak frequency and peak frequency of the lowest note were measured automatically, and all other measurements were performed manually in RavenPro 1.4 [21] using a Hamming window and a fast Fourier transformation (FFT) length of 1024, resulting in a spectral resolution of 47 Hz. Minimum and maximum frequencies were measured using precise placement of a selection box on power spectra at the margin of notes, and placement was verified using the spectrogram view. Mean values of vocal features were calculated for each individual male. For three species, the black-chinned hummingbird (*Archilochus alexandri*), common poorwill (*Phalaenoptilus nuttallii*), and piñon jay (*Gymnorhinus cyanocephalus*), we only recorded a single vocalization for each; therefore, we used vocalizations archived at the Cornell University Macaulay Library (http://macaulaylibrary.org/index.do; catalog numbers 6112, 6113, 20580, 21254, 44632, 44979, 60118, 60120, 60122, 60123, 60124, 109034, 109095, 109113, 119406, 147569) and Xeno-canto (http://www.xeno-canto.org/; catalog numbers XC11631, XC21431, XC21752, XC70461) to increase the number of individual samples for these species. We measured songs for all songbirds (Order Passeriformes), except for the piñon jay, western scrub-jay (*Aphelocoma californica*), and bushtit (*Psaltriparus minimus*), of which common calls were measured. We measured common calls for all non-songbirds. For the 30 species considered here, a mean of 15.17±2.33 SE (min = 5, max = 55) individuals were sampled per species to describe a typical species-specific vocalization in our study area.

Because urban-adapted birds may be predisposed to occupy noisy areas [10], we also used species classifications as urban-tolerant or non-urban as a categorical explanatory variable for bird responses to noise in our non-urban study area. Birds were classified as urban-tolerant or non-urban as found in Hu and Cardoso [10] or Bonier et al. [11], and for species not considered in those studies, we classified species using the criteria used by Hu and Cardoso [10]. Here, “urban-tolerant” reflects species known to breed in urban environments, but they are not necessarily urban specialists. Thirteen species were classified as urban-tolerant, and 17 were classified as non-urban.

### Analyses

Because vocal features were highly correlated, we used principal components analysis (PCA) to reduce log-transformed frequency and duration measures to fewer explanatory variables. These data were suited for reduction (Kaiser-Meyer-Olkin measure of sampling adequacy: 0.68; Bartlett's test of sphericity, χ^2^ = 53.41, d.f. = 5, *p*<0.001). PCA yielded two components with eigenvalues greater than 1 and collectively explained 89.11% of the total variance in the data (Table 1). The first principal component was negatively associated with all four measures of frequency (henceforth “PC_Freq_”). The second principal component was negatively associated with vocalization length and number of notes (henceforth “PC_Dur_”). The scores for PC_Freq_ and PC_Dur_ were then used as composite measures of signal frequency and signal duration, respectively, in subsequent analyses.

**Table 1:** Factor loadings on two principal components for acoustic measures taken from bird vocalizations.

	Factor loadings	
	PC_Freq_	PC_Dur_
Eigenvalue	1.864	1.368
Percent variance	57.926	31.186
Peak frequency	−0.515	
Lowest note peak frequency	−0.493	0.173
Minimum frequency	−0.471	0.101
Maximum frequency	−0.498	0.106
Song length		−0.700
Number of notes	−0.148	−0.677

A blank value indicates that the variables did not load strongly on that principal component axis.

We used generalized linear models (GLMs) to investigate the effects of PC_Freq_, PC_Dur_ and urban-tolerance classification on species' nesting and abundance responses to noise. For our model selection procedure, we used an information-theoretic approach to evaluate support for competing candidate models with Akaike's Information Criterion corrected for small sample sizes (AIC*_c_*) [22]. We ranked models based on differences in AIC*_c_* scores (ΛAIC*_c_*). Models with ΛAIC*_c_*<4 were considered to have support and assigned Akaike weights (*w_i_*). When more than one model received support (ΛAIC*_c_*<4), we used Akaike weights to calculate model-averaged variable coefficient estimates, unconditional standard errors (SE) and 95% confidence intervals (95% CIs). We concluded that there was little evidence for the effect of an explanatory variable on response to noise when the 95% CIs included or overlapped zero. We also reran these analyses at the genus level and once restricted to songbirds (Passeriformes). In all cases, the results remained equivalent to those presented for the full datasets and are not presented here.

In birds, vocal features often co-vary with body size [23]–[26]; therefore, we used linear regression to relate PC_Freq_ and PC_Dur_ to the natural log of body mass and concluded there was evidence for a relationship between body mass and vocal features if the 95% CIs of the coefficient estimates did not overlapped zero. This was necessary in order to compare our findings to well known relationships between body size and species' traits that may influence sensitivity to noise (see discussion). Body mass data were gathered from The Birds of North America Online [27]. Finally, we used two-sample *t*-tests to determine whether vocal features differed between urban-tolerant and non-urban birds. We also compared body mass between these groups to determine whether any differences in vocal features could be explained by differences in body size. All analyses were completed in program R [28].

## Results

**Figure 3:**
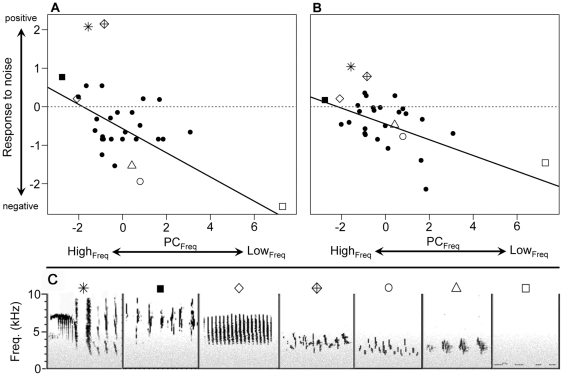
Influence of vocal frequency (PC_Freq_) on response to noise. PC_Freq_ (negatively associated with four vocalization frequency features) had a strong effect on species' (**A**) nesting and (**B**) abundance responses to noise (both panels, *n* = 30). Y-axis values reflect the natural log of the ratios reflecting response to noise: (**A**) mean number of nests per treatment vs. control site and (**B**) mean number of individuals per survey location on treatment vs. control sites. Values above zero (dashed horizontal lines) indicate greater abundance on treatment sites (positive response to noise), and values below zero indicate greater abundance on control sites (negative response to noise). Distance from zero reflects the relative strength of the response. (**C**) Sample spectrograms of species vocalizations (black) and anthropogenic noise with decreasing acoustic energy at higher frequencies (grey; included for display only). For all panels, symbols other than solid circles are as follows: asterisk = black-chinned hummingbird, solid square = bushtit, open diamond = chipping sparrow, crossed diamond = house finch, open circle = black-headed grosbeak, open triangle = western tanager, open square = mourning dove (see also Audio S3, S4, S5, S6, S7, S8, S9 for samples of each species).

Candidate models with PC_Freq_ and the urban-tolerance classification received the most support from both the nesting and abundance data sets and received clear support over null models (Table 2). However, models including PC_Dur_ were also among those with support (ΛAIC*_c_*<4; Table 2). Yet among the model-averaged coefficient estimates, PC_Freq_ had a strong effect on the nesting and abundance responses to noise (Fig. 3), but there was no support for the influence of PC_Dur_ and urban-tolerance classification on either response to noise because the 95% CIs overlapped zero (Table 3, Fig. 3). The negative relationship between PC_Freq_ and response to noise reflected that species with lower frequency vocalizations (including frequencies <2.0 kHz), such as the western tanager (*Piranga ludoviciana*), black-headed grosbeak (*Pheucticus melanocephalus*), and mourning dove (*Zenaida macroura*), had strong negative responses to noise, but species with higher frequency vocalizations (primarily>3.0 kHz), such as the chipping sparrow (*Spizella passerina*), tended to have neutral responses to noise. Still, other species with high-frequency vocalizations, such as the house finch (*Carpodacus mexicanus*), black-chinned hummingbird, and bushtit, tended to respond positively (Fig. 3).

**Table 2:** Model-selection results for full dataset examining the influence of vocal features and urban classification in explaining responses to noise.

Candidate models	*K*	AIC*_c_*	ΔAIC*_c_*	*w_i_*
**Nesting response**				
PC_Freq_, urban	4	79.590	0.000	0.51
PC_Freq_	3	81.050	1.460	0.24
PC_Freq_, PC_Dur_, urban	5	82.049	2.459	0.15
PC_Freq_, PC_Dur_	4	82.758	3.168	0.10
Null	2	89.211	9.621	
Urban	3	90.730	11.140	
PC_Dur_	3	91.010	11.420	
PC_Dur_, urban	4	92.960	13.370	
**Abundance response**				
PC_Freq_, urban	4	53.650	0.000	0.51
PC_Freq_	3	58.302	1.181	0.28
PC_Freq_, PC_Dur_, urban	5	58.504	2.845	0.12
PC_Freq_, PC_Dur_	4	60.929	3.526	0.09
Null	2	64.011	9.643	
Urban	3	64.030	11.290	
PC_Dur_	3	66.450	11.890	
PC_Dur_, urban	4	66.690	13.860	

PC_Freq_ was negatively associated with signal frequency, PC_Dur_ was negatively associated with signal duration, and urban reflects species classification as urban-tolerant (breeding in urban areas) or non-urban. All candidate models are shown, including the null (intercept only model). *K* represents the number of parameters in the model, AIC*c* values are Akaike's information criteria for small sample size and ΛAIC*_c_* is the difference in AIC*_c_* values from the top-ranking model. Models with ΛAIC*_c_*<4 are considered to have support and used to calculate Akaike weights (*w_i_*) for model-averaging coefficient estimates.

**Table 3:** Estimates for the influence of explanatory variables on responses to noise.

Model set/explanatory variables	effect size ± SE	lower, upper CIs
**Nesting response**		
PC_Freq_	−0.314±0.085	−0.487, −0.140^a^
PC_Dur_	0.022±0.069	−0.118, 0.161
urban status – tolerant	0.397±0.384	−0.370, 1.160
**Abundance response**		
PC_Freq_	−0.205±0.055	−0.317, −0.093^a^
PC_Dur_	0.006±0.036	−0.068, 0.079
urban status – tolerant	0.242±0.245	−0.248, 0.733

GLM model-averaged coefficient estimates, plus unconditional standard errors (SE), and lower and upper 95% confidence intervals (CIs) are presented for all explanatory variables in supported models (ΛAIC*_c_*<4).

a Effects with confidence intervals that do not overlap zero, indicating a strong effect.

**Figure 4:**
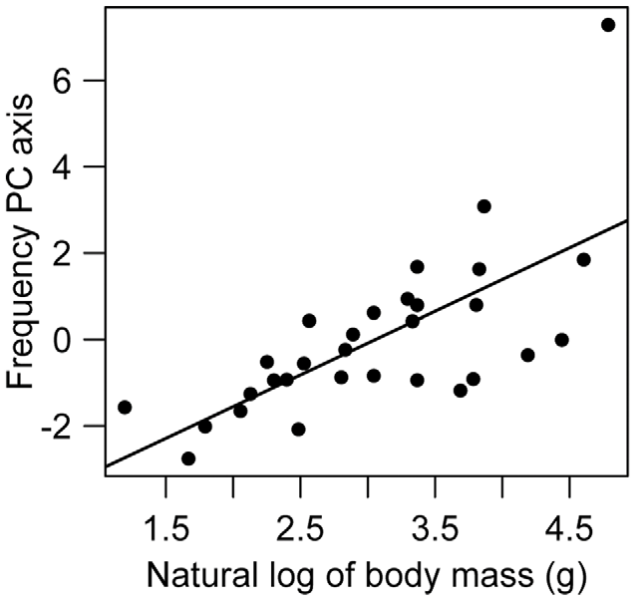
Relationship between body mass and PC_Freq_. Because frequency features are negatively associated with PC_Freq_, the positive relationship depicted reflects a negative relationship between body mass and vocal frequency.

There was no evidence for an influence of body mass on PC_Dur_ values (β_Mass_ = 0.074±0.293, 95% CI = −0.527, 0.675). However, there was a strong positive influence of body mass on PC_Freq_ (β_Mass_ = 1.470±0.288, 95% CI = 0.880, 2.059; Fig. 4), supporting previous findings that frequency is negatively related to body size [23]–[26] and suggesting that larger birds with lower frequency signals may be more sensitive to noise than smaller birds with signals located at higher frequencies. In contrast, neither vocal features nor body mass differed between urban-tolerant and non-urban species (PC_Freq_, two-tailed-t_28_ = 1.150, p = 0.260; PC_Dur_, two-tailed-t_28_ = 0.990, p = 0.331; log*_e_* body mass, two-tailed-t_28_ = 0.659, p = 0.515), suggesting no differences in signal duration, signal frequency or body mass between the two classifications.

## Discussion

Our finding that signal frequency (PC_Freq_) explained variation in responses to noise for two data sets provides evidence for a causal relationship between sensitivity to noise and vocal frequency. In contrast, increased signal duration (PC_Dur_), which may improve signal detection in noisy environments [17]–[19], and urban tolerance failed to explain responses to noise. Although there may be a link between vocal frequency and sensitivity to noise, the relationship should only explain negative or neutral responses to noise in terms of habitat use. It is less clear why smaller species with high-frequency vocalizations responded positively to noise. These positive responses to noise likely depend on species' abilities to successfully dispatch critical signals, but also may represent habitat selection and use based on other cues. For example, in our study area species richness is lower in noisy areas and a key nest predator is less abundant than in quiet areas [6]. It is possible that some species recognize cues indicative of lower interspecific competition or lower nest predation risk and preferentially settle in noisy areas.

Two previous studies support our finding that vocal frequency may influence sensitivity to noise [7], [29]; however, their findings had been somewhat limited due to complications associated with road noise and lack of repetition in the study [7] or due to a small sample of species [29]. However, taken collectively with our findings, they suggest that higher frequency signals are important for species persistence in noisy environments and are in line with the mounting number of studies suggesting that some birds regularly inhabiting noisy areas sing at a higher frequency to reduce masking by noise (e.g., refs. [8], [19], [20], [30], [31], but see ref. [32] for a non-adaptive explanation for frequency changes in noise) and that higher frequency songs are advantageous for male-female communication under noisy conditions [31]. Whether species-specific frequency features and noise-dependent signal adjustments interact, permitting species to remain in noisy environments, is not yet known, but may depend on the degree of spectral overlap between the signal and background noise. For example, noise-dependent increases in signal frequency are often fairly small (approximately 200 to 900 Hz) [8], [19], [20], [30], [32]; therefore, frequency increases for species with low-frequency signals may not increase the contrast between the signal and noise because noise may have considerable energy at frequencies well above low-frequency signals. Instead, species with low-frequency vocalizations may need to rely on other noise-dependent vocal adjustments or abandon noisy areas.

One limitation of our study was that we were unable to examine the influence of vocal amplitude on different species' responses to noise because of the many complications associated with measuring vocal amplitude in free-living birds [32], [33]. Communication theory and empirical data support the notion that signaling with greater amplitude increases signal detection by a receiver [17], [18], [32]. Additionally, the increase in vocal amplitude in response to noise (the Lombard effect) appears to be a widespread strategy employed by birds and mammals to overcome noisy signaling conditions (reviewed in ref. [34]), and it is probable that individuals included in this study were responding to noise exposure with increases in vocal amplitude.

Despite potential increases in amplitude by individual birds vocalizing in noise, species-specific differences in vocal amplitude could also affect their sensitivities to masking by noise. Yet because avian body mass is positively related to vocal amplitude [35] or loudness [24], but negatively related to vocalization frequency [23], [25], [26], expectations of how vocal amplitude and frequency trade-off to affect vocal communication in areas with low-frequency noise is less clear. On one hand, the ability to effectively communicate should increase with body size via higher signal amplitudes. On the other hand, communication should become progressively more difficult with increases in body size due to decreases in signal frequency. Although we did not explicitly test for an influence of vocal amplitude, we found a strong negative relationship between body mass and PC_Freq_. This implies that higher vocal amplitudes of larger species may not be sufficient to overcome the masking potential of noise, but the frequency content of the signal may be more important. That is, larger birds may be able to vocalize more loudly, but they also vocalize at lower frequencies where noise has more acoustic energy. This problem for larger birds may be further compounded by their defense of larger territories [36], whereby communication distances are greater between individuals.

Although noise may exclude species with low-frequency vocalizations from noisy environments, this does not necessarily mean there are no costs for those that remain. Many of the negative nesting responses to noise were stronger than the negative abundance responses, which could represent a greater proportion of unpaired males on treatment sites relative to control sites, a pattern previously observed for reed buntings (*Emberiza schoeniclus*) and ovenbirds (*Seiurus aurocapilla*) breeding in noisy and quiet areas [30], [37]. Whether patterns of pairing success within noisy areas depend on the degree to which males' signals are masked by low-frequency noise is unknown; however, within established pairs, masking can impair male-female communication by masking low-frequency songs that are preferred by female great tits (*Parus major*) [31]. Masking of low-frequency signals that are reliable cues of male quality and condition [38], [39] could also explain patterns of reduced clutch sizes for great tits nesting in noisy areas, whereby females' song-based assessments of male quality are compromised and females invest less energy in egg production [40]. It is also possible that masking of low-frequency signals could compromise females' abilities to discriminate among males, leading to maladaptive mating decisions by pairing with smaller or lower quality males whose higher frequency signals are masked less by noise. Key to understanding the full costs of breeding in noisy areas will require studies that integrate data on individual pairing success, body size, and signal features.

Previous findings suggest that urban birds are predisposed to noisy conditions with higher frequency songs [10], yet we did not find vocal features to differ between urban-tolerant and non-urban birds, nor did body mass or response to noise differ between these groups. One potential explanation for these conflicting results is that we did not use within-genus species pairs as did Hu and Cardoso [10]; however our study had the advantage of examining species-specific responses to noise in the absence of corollaries of urbanization; thus, we were limited to the species that regularly breed in our study region. Regardless of urban-tolerance classification, we found that most species tended to respond negatively to noise. This suggests that even urban-adapted species that may have broad tolerances to a variety of environmental conditions may still be sensitive to noise. Instead, their ubiquity in urban areas may depend more on access to foraging and nesting resources [12] or potentially depend on the absence of key predators or competitors that avoid urban areas [6]. Still needed are studies that aim to understand how these forces interact with noise exposure to influence settlement and habitat use patterns within cities.

Our findings provide strong evidence that chronic noise filters bird communities by masking acoustic communication and strengthens the growing body of evidence that human-generated acoustics represent a selective force shaping the ecology of birds in noisy landscapes. Those species most likely to abandon noisy areas are birds with low-frequency signals, which also tend to have larger bodies. In contrast, smaller species may not only persist in noisy environments through transmission of higher frequency signals, but benefit from increased reproductive success relative to those nesting in less noisy areas due to reduced predation risk [6]. Yet the benefit associated with reduced predation may be a fitness tradeoff balanced by costs related to male-female communication, pairing success, and reproductive success in the absence of predation [30], [31], [37], [40]. Given that increases in noise exposure is a global phenomenon, more attention is needed to evaluate individual and population-level tradeoffs associated with breeding in noisy areas, even among urban-tolerant species that may also respond negatively to noise. At the community-level, we must still determine whether noise is an agent of ecological filtering for other taxa that rely on acoustic communication.

## Supporting Information

Audio S1 **Sample recording of background noise on a treatment site at a distance of 100 m from the compressor exhaust.** See Fig. 2 in the main text for spectrogram and power spectra displaying the distribution of acoustic energy.
(WAV)

Audio S2 **Sample recording of background noise on a control site at a distance of 100 m from the natural gas wellhead.** See Fig. 2 in the main text for spectrogram and power spectra displaying the distribution of acoustic energy.
(WAV)

Audio S3 **Sample recording of black-chinned hummingbird vocalizations. See** **Fig. 3(C)** **in main text for spectrogram.**
(WAV)

Audio S4 **Sample recording of bushtit vocalizations.** See Fig. 3(**C**) in main text for spectrogram.
(WAV)

Audio S5 **Sample recording of chipping sparrow vocalizations.** See Fig. 3(**C**) in main text for spectrogram.
(WAV)

Audio S6 **Sample recording of house finch vocalizations.** See Fig. 3(**C**) in main text for spectrogram.
(WAV)

Audio S7 **Sample recording of black-headed grosbeak vocalizations.** See Fig. 3(**C**) in main text for spectrogram.
(WAV)

Audio S8 **Sample recording of western tanager vocalizations.** See Fig. 3(**C**) in main text for spectrogram.
(WAV)

Audio S9 **Sample recording of mourning dove vocalizations.** See Fig. 3(**C**) in main text for spectrogram.
(WAV)
